# Ten Years for GHSP: Where Are We Now? Where Will We Go?

**DOI:** 10.9745/GHSP-D-23-00102

**Published:** 2023-04-28

**Authors:** Zulfiqar A. Bhutta, Rajani Ved, Nana Twum-Danso, Abdulmumin Saad, Stephen Hodgins

**Affiliations:** a Dalla Lana School of Public Health, University of Toronto, Toronto, Canada; Centre for Global Child Health, Hospital for Sick Children, Toronto, Canada; Centre for Excellence in Women and Child Health and Institute of Global Health and Development, The Aga Khan University, Karachi, Pakistan.; b Bill & Melinda Gates Foundation, New Delhi, India.; c Institute for Healthcare Improvement, Boston, MA, USA.; d Bill & Melinda Gates Foundation, Washington, DC, USA.; e Editor-in-Chief, Global Health: Science and Practice Journal; Associate Professor, School of Public Health, University of Alberta, Edmonton, Alberta, Canada.

In 2013, *Global Health: Science and Practice* (GHSP) launched with funding from the U.S. Agency for International Development. From the beginning, GHSP’s vision was to leverage knowledge gleaned from the implementation of health programs and services to improve programs and, ultimately, health outcomes and well-being. Shortly after GHSP’s launch, in 2016, the world transitioned from the Millennium Development Goals to the more wide-ranging Sustainable Development Goals.^1^

## A CHANGING LANDSCAPE

Over the past decade, global funding agencies and initiatives, including Gavi, the Global Fund, and the World Bank’s Global Financing Facility, have served as accelerators for change in various areas, including immunization, HIV, malaria, as well as maternal, newborn, child, and adolescent health. These new models of development financing have helped bridge gaps in some of the world’s poorest countries.^2^^,^^3^ But they have also contributed to the verticalization of program efforts at the global and often country level, with limited integration even within programs. For example, in many countries, there has been little integration between polio eradication efforts and the delivery of routine immunization services.^4^

Since 2020, when the COVID-19 pandemic began, the world has faced numerous health crises, natural disasters, and conflicts that have had devastating health and economic consequences worldwide, reversing progress on achieving the Sustainable Development Goals.^5^^,^^6^ According to the World Bank, the pandemic pushed about 70 million people into extreme poverty in 2020, the largest 1-year increase since global poverty monitoring began in 1990.^7^ Recovery has been uneven. Because of the pandemic, many populations have experienced important indirect effects, including adverse impacts on health outcomes due to the disruptions in health services and mental health effects arising from physical distancing and school closures, among others.^8^ These impacts were particularly experienced by vulnerable populations in low- and middle-income countries (LMICs). The pandemic also laid bare fundamental weaknesses in national, regional, and global provisions for emergency prevention, preparedness, and response.

These challenges, as important as they are, are dwarfed by the almost existential threat that climate change poses to global health and development, with impacts on nutrition and health outcomes,^9^^–^^11^ as well as on the redirection of development funds and human resources to address climate shocks, natural disasters, and changing weather patterns.

## WHAT CAN BE DONE?

Given these multidimensional threats, achieving the Sustainable Development Goals will require a serious collaborative effort globally. In every country and region, there needs to be a robust and transparent analysis of gaps and areas where there has been regression or stagnation, as well as greater advocacy with global policymakers and funders to regain lost momentum to address persisting challenges and strengthen health and nutrition systems. The interlinked strategies for universal health coverage and primary care have been highlighted by member countries of the World Health Assembly before the pandemic; once again, they need to become central to planning.

Moving forward over the coming decade, global health will require astute and focused efforts to address these and future complex challenges within a shrinking fiscal space for large-scale programs. All stakeholders, including funders and governments, must work together in a coordinated fashion, consistent with each country’s national adaptation plans, to address implementation challenges. In doing so, these complex challenges can be managed more effectively and efficiently—an essential requirement in settings where financial and human resources are severely constrained. This, in turn, will require resources and political support within countries and prioritization by global funding bodies. Global leaders have, in the past, demonstrated an ability to rise to the occasion, as evidenced by record levels of development assistance during the pandemic.^12^ Mobilizing resources and public support for global health will require increased political commitment and funding. As underscored by Pablo-Mendez,^13^ at the global level, development assistance is now refocusing on fragile states, the poorest communities, and cooperation on global public goods, such as health security, technical norms, and innovation. Countries need to focus on leveraging domestic finances and supporting priority-setting and decision-making with local engagement and ownership.

This year, at GHSP, we want to encourage reflection and discussion on key issues we believe should be prioritized over the coming decade. We invite submissions (in the form of viewpoints and commentaries) focusing particularly on the following issues.

### Universal Health Coverage

Addressing the structural and systemic inequalities that affect health care access, quality, and coverage is key to achieving universal health coverage. The determinants of inequity vary across contexts, and it is critical for governments across the world to take urgent action. We need to refocus attention on reaching the most marginalized and reducing inequities by addressing social and commercial determinants of health.^14^^,^^15^ Not only does this call for attention to the required social safety nets and information systems but also to effective service delivery and human resources capable of delivering high-quality primary care. While digital technology for health has enabled improvements in patient care delivery, outcomes, and access, unequal access to Internet connectivity and devices has created a new form of inequity—the digital divide. A better understanding of policy and practice related to digital technology is required to address this form of inequity. Artificial intelligence technology is rapidly evolving and has the potential to help overcome barriers to health care access and quality if adopted in line with local needs, priorities, and contexts.

### Mental Health

Mental health disorders, which already present a significant global disease and economic burden, have been exacerbated by the COVID-19 pandemic,^16^ as well as other crises emerging since then, and warrant a prominent place on the global health agenda over the coming decades.

### Other Noncommunicable Diseases

Existing health programs have generally focused on specific infectious diseases, ignoring major emerging global challenges, notably noncommunicable diseases.^17^ This neglect contributed to significant excess mortality during the COVID-19 pandemic, especially among marginalized populations with limited access to care.^18^ As populations in LMICs become more urbanized and sedentary, more attention will need to be focused on noncommunicable diseases of public health importance, such as cancers, hypertension, and nutrition-related disorders, including obesity, diabetes, and cardiovascular diseases.

### Sexual and Reproductive Health Care

More than 1 million additional maternal deaths are estimated by 2030 on current trends.^19^ If all women in LMICs wanting to prevent a pregnancy were to use modern contraceptives and all pregnant women and their newborns were to receive quality health care services, unintended pregnancies, unsafe abortions, and maternal deaths would substantially decrease.^20^ Investment in universal access to family planning, including integration with reproductive and maternal health services, is an urgent priority in LMICs.

### Climate Change

The Lancet Countdown, a collaboration between 24 academic institutions and intergovernmental organizations, projects that the impacts of climate change will worsen with time and are likely to have major deleterious effects on health.^21^ Building resilience against current and emerging global challenges of climate change should become a national priority across all countries. This requires stepping back from a narrow focus on pandemic preparedness and embracing a broader interconnected agenda promoting multisectoral action.

## CONCLUSION

GHSP’s initial vision of serving as a platform to disseminate robust evidence from real-world program implementation is as relevant today as it was a decade ago. By sharing knowledge gleaned by those engaged in global health programs, GHSP will continue to help the field of global health integrate practical knowledge to adapt and better address present challenges, as well as prepare for future needs and priorities toward making sustainable change.

## Notes

**First published online:** April 13, 2023.
